# Supplementary material for: Patient-specific Boolean models of signalling networks guide personalised treatments
Source: eLife. 2022 Feb 15;11:e72626. doi: 10.7554/eLife.72626 (PMC9018074; doi:10.7554/eLife.72626)
Supplement: Transparent reporting form [file elife-72626-transrepform1.docx]

***eLife’s* transparent reporting form**

We encourage authors to provide detailed information *within their submission* to facilitate the interpretation and replication of experiments. Authors can upload supporting documentation to indicate the use of appropriate reporting guidelines for health-related research (see [EQUATOR Network](http://www.equator-network.org/%20)), life science research (see the [BioSharing Information Resource](https://biosharing.org/)), or the [ARRIVE guidelines](http://www.plosbiology.org/article/info:doi/10.1371/journal.pbio.1000412) for reporting work involving animal research. Where applicable, authors should refer to any relevant reporting standards documents in this form.

If you have any questions, please consult our Journal Policies and/or contact us: [editorial@elifesciences.org](mailto:editorial@elifesciences.org).

**Sample-size estimation**

- You should state whether an appropriate sample size was computed when the study was being designed
- You should state the statistical method of sample size computation and any required assumptions
- If no explicit power analysis was used, you should describe how you decided what sample (replicate) size (number) to use

Please outline where this information can be found within the submission (e.g., sections or figure legends), or explain why this information doesn’t apply to your submission:

LNCaP cells were used in the latter part of the study by doing endpoint cell viability measurements (assay 1) and real-time cell electronic sensing (RT-CES) cytotoxicity assay (assay2). Over 10000 LNCaP cells per well were studied in the assay 1 and 20000 cells per well in the assay 2. All this information is available in the Material and methods section and results of these assays can be seen in Figures 7 for the assay 1 and Figures 8 and 9 for the assay 2.

No explicit power analysis was used to compute the number of cells (sample size).

**Replicates**

- You should report how often each experiment was performed
- You should include a definition of biological versus technical replication
- The data obtained should be provided and sufficient information should be provided to indicate the number of independent biological and/or technical replicates
- If you encountered any outliers, you should describe how these were handled
- Criteria for exclusion/inclusion of data should be clearly stated
- High-throughput sequence data should be uploaded before submission, with a private link for reviewers provided (these are available from both GEO and ArrayExpress)

Please outline where this information can be found within the submission (e.g., sections or figure legends), or explain why this information doesn’t apply to your submission:

LNCaP cells were used in the latter part of the study by doing endpoint cell viability measurements (assay 1) and real-time cell electronic sensing (RT-CES) cytotoxicity assay (assay2). Two replicates were studied in each assay, except for PI-103 in 1nM in assay 1 and the 3.3uM treatments in assay 2. This has been stated in the Material and methods section.

There is an outlier datapoint at time 16 hours in the assay 2 (Figures 8A, 8E, 9A and 9E) but this was not excluded as these data were used to validate qualitatively the effect of drugs, but no statistical analysis was applied on it.

Results of these assays can be seen in Figures 7 for the assay 1 and Figures 8 and 9 for the assay 2.

**Statistical reporting**

- Statistical analysis methods should be described and justified
- Raw data should be presented in figures whenever informative to do so (typically when N per group is less than 10)
- For each experiment, you should identify the statistical tests used, exact values of N, definitions of center, methods of multiple test correction, and dispersion and precision measures (e.g., mean, median, SD, SEM, confidence intervals; and, for the major substantive results, a measure of effect size (e.g., Pearson's r, Cohen's d)
- Report exact p-values wherever possible alongside the summary statistics and 95% confidence intervals. These should be reported for all key questions and not only when the p-value is less than 0.05.

Please outline where this information can be found within the submission (e.g., sections or figure legends), or explain why this information doesn’t apply to your submission:

Exact p-values have been reported in the section “Drugs associated with the proposed targets” of the main text and in the section “Drugs associated to genes included in the model” in the Appendix supplementary file.

(For large datasets, or papers with a very large number of statistical tests, you may upload a single table file with tests, Ns, etc., with reference to sections in the manuscript.)

**Group allocation**

- Indicate how samples were allocated into experimental groups (in the case of clinical studies, please specify allocation to treatment method); if randomization was used, please also state if restricted randomization was applied
- Indicate if masking was used during group allocation, data collection and/or data analysis

Please outline where this information can be found within the submission (e.g., sections or figure legends), or explain why this information doesn’t apply to your submission:

LNCaP cells were treated with drugs in the latter part of the study by doing endpoint cell viability measurements (assay 1) and real-time cell electronic sensing (RT-CES) cytotoxicity assay (assay2).

We assigned randomly the 96-wells to the 4 different drug treatments (5 concentrations and a control for assay 1 and 3 concentrations and a control for assay 2).

**Additional data files (“source data”)**

- We encourage you to upload relevant additional data files, such as numerical data that are represented as a graph in a figure, or as a summary table
- Where provided, these should be in the most useful format, and they can be uploaded as “Source data” files linked to a main figure or table
- Include model definition files including the full list of parameters used
- Include code used for data analysis (e.g., R, MatLab)
- Avoid stating that data files are “available upon request”

Please indicate the figures or tables for which source data files have been provided:

As source data and source code and in the code repository that accompanies this work (<https://github.com/ArnauMontagud/PROFILE_v2#scripts-to-reproduce-figures-of-the-paper>), we have provided processed data and code to reproduce figures 4-9. Figure 1 is a graphical abstract of the work, Figure 2 can be obtained from Appendix 2 and Figure 3 from Appendix 3.
